# Nonclinical safety evaluation of food colorant lac dye *via* systematic toxicity profiling with assessment of *in vivo* antigenic potential

**DOI:** 10.3389/fphar.2022.1020379

**Published:** 2022-10-31

**Authors:** Jin-Sung Park, Seung-Hyun Kim, Yun-Soon Kim, Euna Kwon, Hyun-Jin Lim, Kang-Min Han, Yang-Kyu Choi, Chul-Woo Jung, Byeong-Cheol Kang

**Affiliations:** ^1^ Department of Experimental Animal Research, Biomedical Research Institute, Seoul National University Hospital, Seoul, South Korea; ^2^ Graduate School of Translational Medicine, Seoul National University College of Medicine, Seoul, South Korea; ^3^ Department of Pathology, Dongguk University Ilsan Hospital, Goyang, Gyeonggi-do, South Korea; ^4^ Department of Laboratory Animal Medicine, College of Veterinary Medicine, Konkuk University, Seoul, South Korea; ^5^ Department of Anesthesiology and Pain Medicine, Seoul National University Hospital, Seoul, South Korea; ^6^ Biomedical Center for Animal Resource and Development, Seoul National University College of Medicine, Seoul, South Korea; ^7^ Designed Animal and Transplantation Research Institute, Institute of GreenBio Science Technology, Seoul National University, Pyeongchang-gun, Gangwon-do, South Korea

**Keywords:** lac dye, laccaic acids, nonclinical safety evaluation, repeated oral toxicity, genotoxicity, antigenicity

## Abstract

Lac dye is a natural colorant derived mainly from the insect *Kerria lacca* (Kerr) and has been used in food and beverage as a red-coloring additive. Despite its increasing use for human consumption as an alternative for allergy-associated cochineal, its toxicity profile remained incomplete to sufficiently assess its safety for the intended use. In this study, we evaluated systemic and genetic toxicity by performing acute and subacute oral toxicity studies in Sprague–Dawley (SD) rats using highly purified lac dye (LD) formulated in water and a battery of genotoxicity tests, respectively. To assess antigenic potentials, we carried out an *in vivo* passive cutaneous anaphylaxis test. A single dose of LD did not cause mortality at 5000 mg/kg body weight (BW), setting oral LD_50_ of >5000 mg/kg BW in SD rats. In the 90-day study, transient salivation without accompanying histopathological lesions in the salivary glands in 200 and 500 mg/kg BW groups and red-purple pigmentation on the surface of femora and skulls in 500 mg/kg groups were observed as nonadverse effects associated with LD, and no adverse effect was detected in all of the parameters examined, establishing a 500 mg/kg BW as no-observed-adverse-effect-level (NOAEL). Furthermore, LD was not mutagenic nor clastogenic in the genotoxicity tests. When tested for antigenicity, LD did not induce anaphylactic skin responses as opposed to the positive reaction by ovalbumin, suggesting a lack of antigenicity. Taken together, these findings provide extended toxicity information on LD with direct evidence supporting the lack of antigenicity, providing essential guidance for its safe use in humans.

## Introduction

Food colorants are increasingly used as an additive to improve consumers’ preference and palatability of food. Since identifying actual and potential risks to human health in several synthetic colorants and the eventual removal from the market, health-related concerns with regard to these synthetic colorants (Amchova et al., 2015) have led to an increasing market preference for natural ones and thereby caused a shift in the food industry’s choice of food colorants from artificial products to naturally sourced alternatives, rapidly increasing the usage of natural colorants.

Lac dye is a natural colorant that originated mainly from the lac insect *Kerria lacca* (Kerr). The insect secrets lac dye along with resinous lac, which provides self-protection by creating encrustation of its body, and the dye is extracted as a by-product during the manufacturing process of shellac, which is mainly used for a quick drying wood finisher. Traditionally, lac dye has been used as a dyeing agent for natural fibers such as wool and cotton using its bright red color and more recently as a food colorant in various products for human consumption. The major constituent of lac dye is laccaic acid, which is classified as a hydroxyl-anthraquinone carboxyl acid and the responsible component rendering its characteristic color. Since the first nomenclature by Schmidt in 1887, five laccaic acid isomers, namely, A, B, C, D, and E, have been identified. Among them, A isomer is known to be the most abundant, while D isomer is rarely found in lac dye likely due to its role as a common substrate for the biosynthesis of other isomers (Shamim et al., 2016).

With a growing number and consumption, a portion of natural colorants were discovered to possess harmful effects that could cause serious health problems in humans; madder color, a yellow coloring agent extracted from the roots of *Rubia tinctorum*, was used as a food additive in Asian countries including Korea and Japan, but it was rescinded from the lists of government-approved food colorants in 2004 due to the carcinogenicity observed in a subacute toxicity and a chronic carcinogenicity studies (Inoue et al., 2009a; Inoue et al., 2009b). Another example is the red-colored cochineal extracted from dried female coccids that has been extensively used in food. Although still being used in the market, this colorant was found to cause an acute and severe form of allergic reaction known as anaphylaxis in children who consumed cochineal-containing food and beverage on several occasions (Wüthrich et al., 1997; Voltolini et al., 2014; de Gier and Verhoeckx, 2018). Similarly, allergic reactions including anaphylaxis have been reported on annatto, an orange–yellow dye derived from the seed of the achiote tree *Bixa orellana* (Nish et al., 1991; Sadowska et al., 2021). These cases raised public concerns about the safety of natural colorants that were initially considered a safer option, requiring a systematic and comprehensive assessment of toxicity for novel and currently accepted colorants to confirm their suitability as food additives.

Lac dye has been increasingly used in food and beverage as an alternative for cochineal due to its comparable color and physicochemical properties compatible for industrial use. Although lac dye is currently listed as a food additive in China, Japan, and Korea and 2 mg/kg/day is set as the acceptable daily intake in India (Francis, 1996; Shin, 2016), its entomological origin, similar to cochineal, made us question whether it is sufficiently safe to be used for such purposes. Several studies have been performed to answer this question, and it has been found that the colorant was relatively safe with none to low toxicity in rodents when fed up to 5% in a diet (Sakamoto et al., 1998; Sakamoto et al., 2000) or a 1000 mg/kg BW (Thombare et al., 2017) and nongenotoxic in in vitro assays (Banerjee et al., 1984). Nonetheless, the potential of lac dye to function as an antigen and thereby cause hypersensitivity has remained unexplored. Additionally, the employment of feed-mixed formats for administration may be applicable to evaluate the test substance added to solid food but not closely simulating the circumstance when the colorant is used in beverages. In the present study, we, therefore, use lac dye suspended in water to directly mimic its use in beverages to evaluate its toxicity. After characterization of the test substance, general systemic toxicity and genotoxicity were assessed by performing single-dose and 90-day repeated-dose toxicity studies and using a battery of genotoxicity tests, respectively. To test the immunogenic potential, we carried out an *in vivo* anaphylaxis test. Our findings provided much-needed information on the toxicity of lac dye for its safe use for human consumption.

## Materials and methods

### Test substance and chemicals

The test article was lac dye (LD; color value E (10%, 1 cm, 490 nm) = 1421) produced by Hefei Light Industrial Product, Arts & Crafts I&E Co. Ltd. (Hefei, China) and purchased through a local importer. A voucher specimen (SNUH 18020-1) has been deposited at the Department of Experimental Animal Research, Biomedical Research Institute, Seoul National University Hospital. All other chemicals and reagents used in this study were purchased from Sigma-Aldrich (MO, United States) unless otherwise stated.

### Test substance analysis

High-performance liquid chromatography (HPLC) was performed to determine the levels of laccaic acids in LD. In brief, natural red 25 (NR25; #50506, Sigma Aldrich, MO, United States) was dissolved in the mobile phase [1:1 mixture of 0.3% trifluoroacetic acid in water (solution A) and 0.3% trifluoroacetic acid in methanol (solution B)] to prepare a 1 mg/ml solution and used after dilution, and the test substance was similarly prepared. After comparing absorbance spectra measured between 440 and 590 nm, 10 μl of the analytical standard and LD sample were separated using a Unison UK C8 (4.6 mm × 250 mm, 3 μm) column in a Nanospace SI-2 (Shiseido, Tokyo, Japan) at 40°C with a speed of 0.8 ml/min using gradient elution (the ratio of solutions A and B; 30:70 for 0–12 min, 32:68 for 12–27 min, 38:62 for 27–27.1 min, and 30:70 for 27.1–35 min).

### Animals

The animals used in this study were obtained from Orient Bio Inc. (Gyeonggi-do, Korea) and housed in environment-controlled animal rooms (temperature at 20–26°C, relative humidity of 50 ± 20%, 12-h light/dark cycle, ventilation 10–15 times/hour, and light intensity 150–300 Lux) at the Association of Assessment and Accreditation for Laboratory Animal Care (AAALAC) International-accredited animal facility (#001160) in Seoul National University Hospital as previously described (Park et al., 2020). Animals were given free access to a γ-irradiated rodent diet (Teklad certified irradiated global 18% protein rodent diet, 2918C, Envigo RSM Inc., IN, United States) and autoclaved water. All experiments were approved by the Institutional Animal Care and Use Committee in Seoul National University Hospital and carried out in a GLP-accredited facility of the institution.

### Oral toxicity study

SD rats (216.42–270.79 g for males and 161.28–208.63 g for females) were acclimated for a week and used for studies investigating acute oral toxicity (*n* = 5/gender/group) and 90-day repeated oral toxicity (*n* = 10/gender/group). An acute oral toxicity study was carried out according to the Organization for Economic Cooperation and Development (OECD) test guideline No. 420 (OECD, 2001). The treatment groups received an oral gavage of either 1250, 2500, or 5,000 mg/kg BW of LD suspended in ddH_2_O once while the vehicle control group had a 15 ml/kg BW of the vehicle. All animals were monitored once in every hour up to 6 h and then once daily for 14 days. Body weight was measured every week. At the end of the study, all animals were sacrificed and examined for gross lesions.

A 90-day repeated oral toxicity study was carried out according to the OECD test guideline No. 408 (OECD, 2018). The treatment groups daily received oral gavages of 50, 100, 200, and 500 mg/kg BW of LD, and the vehicle control group 10 ml/kg BW of the vehicle for 90 days. During the study period, all animals were monitored daily for clinical signs with weekly measurements of body weight, food, and water consumption. Urinalysis and ophthalmological examination were performed on the half of animals per group in the last week of the study. Regarding urinalysis, each animal’s urine was freshly collected on a clean glass plate and immediately analyzed using Multistix^®^ 10 SG Reagent strips (Siemens Healthineers, Germany) and a CLINITEK Advantus analyzer (Siemens Healthineers). A veterinarian carried out the ophthalmological examination by gross examination and fundoscopy.

### Whole blood and serum analysis

All surviving animals from the 90-day repeated toxicity study were subjected to blood collection under deep anesthesia using isoflurane and then necropsy after euthanasia by exsanguination. Ethylenediaminetetraacetic acid (EDTA)-treated whole blood collected from the vena cava was analyzed using an ADVIA2120i animal blood counter (Siemens Healthcare Diagnostics Ltd., Ireland) for several parameters: red blood cells (RBC), reticulocytes, platelets, white blood cells and each white blood cell type (neutrophils, eosinophils, basophils, lymphocytes, and monocytes), hemoglobin (HGB), hematocrit (HCT), mean corpuscular volume (MCV), mean corpuscular hemoglobin (MCH), and mean corpuscular hemoglobin concentration (MCHC). Blood coagulation ability was assessed by measuring partial thromboplastin time (PT) and activated partial thromboplastin time (aPTT) using an ACL 100 coagulation analyzer (Instrumentation Laboratory, United States)

Serum was separated from the coagulated whole blood by centrifugation and analyzed using a Hitachi 7180 automatic chemistry analyzer (Hitachi Ltd., Japan) to determine the levels of blood urea nitrogen (BUN), total cholesterol (TC), high- and low-density lipoprotein cholesterol (HDLC and LDLC), total protein (TP), albumin, alkaline phosphatase (ALP), aspartate transaminase (AST), alanine transaminase (ALT), γ-glutamyl transferase (GGT), creatinine, triglyceride (TG), glucose, albumin/globulin ratio (A/G), potassium, chlorine, sodium, calcium, and phosphorus.

### Histopathological examination

All major organs were examined for gross lesions at necropsy and their wet weight measured before submerging into appropriate fixatives; Testes and epididymides were fixed in Bouin’s solution, the eyes and Harderian glands in Davidson’s solution, and all other organs in 10% neutral buffered formalin. Femora and nasal cavities were decalcified after fixation. A portion of each fixed organ was dissected out and paraffinized after dehydration. A 2–3 μm section was made from each organ using a microtome and was examined for microscopic legions by two pathologists after staining with hematoxylin and eosin and then peer-reviewed.

### Bacterial reverse mutation test

Mutagenicity of LD was assessed using the bacterial reverse mutation test following the OECD test guideline No. 471 (OECD, 1997). After testing cytotoxicity using *Salmonella typhimurium* TA100 tester strain, one dose among 0, 312.5, 625, 1250, 2500, and 5000 μg/plate of LD was treated to two sets of *S. typhimurium* tester strain TA98, TA100, TA1535, and TA1537 and *Escherichia coli* tester strain WP2*uvrA* (Moltox, United States) in triplicate after preincubation at 37°C for 30 min. LD treated in the second set was metabolically activated using an S-9 mixture (Orient Yeast Co. Ltd., Japan), while the same volume of phosphate buffer (pH 7.4) was used for the first set. The capacity of the S-9 mixture in metabolic activation was confirmed using *S. typhimurium* tester strain TA1538. The following chemicals were used as positive controls for each tester strain; for the second set with metabolic activation, 2-aminoanthracene was used at 2.0 μg/plate for TA98 and TA100 or 5.0 μg/plate for TA1535, TA1537, and WP2*uvrA*, while 10.0 μg/plate 2-nitrofluorene for TA98, 5.0 μg/plate sodium azide for TA100, 0.5 μg/plate SA for TA1535, 80.0 μg/plate 9-aminoacridine for TA1537, and 2.5 μg/plate methylmethane sulfonate for WP2*uvrA* in the absence of an S-9 mixture. All plates were incubated for 48 h at 37°C after treatment and the number of revertant colonies were counted. All experiments using genetically modified bacterial tester strains were approved by the Institutional Biosafety Committee in Seoul National University Hospital.

### *In vitro* chromosome aberration test

*In vitro* clastogenicity of LD was assessed using Chinese hamster lung (CHL) cells following the OECD test guideline No. 473 (OECD, 2014). Initially, the cytotoxicity of LD was tested by determining relative population doubling (RPD) under three different conditions, 1) 6-h treatment, 2) 24-h treatment, and 3) 6-h treatment with the S-9 mixture, and determine the highest dose for each condition. In the main study, the highest dose and lower two doses that were prepared by two-time serial dilution were treated on CHL cells in two flasks: 375, 750, and 1500 μg/ml for 6-h treatment; 125, 250, and 500 μg/ml for 24 h; and 625, 1250, and 2500 μg/ml for 6 h with the S-9 mixture. Positive control groups were treated with 0.1 μg/ml mitomycin C for treatment without the S-9 mixture, while 5.0 μg/ml cyclophosphamide was used for co-treatment with the S-9 mixture. For conditions 1) and 3), the cells were incubated further for 18 h after replacing the media with flesh media after the 6-h treatment, and 0.2 μg/ml colcemid was added to the media of all plates at 2 h before completion of the treatment. The cells were then collected and resuspended in 37°C 0.075 M KCl. After fixation by adding a cold 3:1 mixture of methanol and glacial acetic acid, the two slides of cells per flask were generated and stained with 4% Giemsa solution. The slides were observed under bright field microscopy and the number of cells containing aberrant chromosomes and their types were recorded from 150 cells per flask (a total of 300 cells per dose).

### *In vivo* micronucleus test

*In vivo* clastogenicity was assessed using the mice micronucleus test in compliance with the OECD test guideline No. 474 (OECD, 2016). The animals used in the study were male ICR mice purchased from Orient Bio Inc. (Gyeonggi-do, Korea) and 8-week-old healthy mice (32.1–33.8 g) were selected after acclimation for 5 days. After determining the maximum tolerated dose (MTD), the animals (*n* = 5/group) received oral administration of either 0, 500, 1000, or 2000 mg/kg BW of LD once a day for 4 days. During the administration, clinical signs and body weight were monitored daily. Mitomycin C (2 mg/kg BW) was used as the positive control and intraperitoneally injected 24 h before sacrifice. All surviving animals were euthanized by cervical dislocation. The bone marrow cells collected from the femora by perfusion with fetal bovine serum were attached onto two slide glasses per mouse and stained with 5% Giemsa solution after treatment with 0.004% citric acid and air-drying. Under a bright field microscope, normochromatic (NCE) and polychromatic erythrocytes (PCE) were identified, and two thousand PCE per slide were examined. The number of micronucleus-positive PCE (MNPCE) was counted. Percentage of PCE [PCE/(NCE + PCE) and percentage of NCE/PCE were also calculated.

### Passive cutaneous anaphylaxis reaction test

The passive cutaneous anaphylaxis test was performed as a non-GLP study to test whether LD was antigenic. Male C57BL/6 mice (*n* = 40; 17.58–21.14 g) and male SD rats (*n* = 80; 426.12–570.88 g) were obtained from Orient Bio Inc. (Gyeonggi-do, Korea). Mice (*n* = 5/group) were sensitized by oral administration of either 0, 0.5, 1.0, 2.0, or 4.0 mg/kg BW of LD three times per week for 3 weeks. An additional group of mice was subcutaneously injected with an emulsified solution of 4 mg/kg BW of LD mixed with Freund’s complete adjuvant (FCA) weekly for 3 weeks, while the positive control group received 5 mg/kg BW of ovalbumin mixed with FCA. All animals were sacrificed under deep anesthesia 14 days after the final administration, and serum was separated from the blood collected from the vena cava. Immunization serum was prepared by two-fold serial dilution in normal saline to be 1/2^1^–1/2^10^ of the original serum and SD rats (*n* = 2/group) were intradermally injected with 0.05 ml of the diluted serum in the back skin. The test substance used for the challenge was prepared by completely dissolving LD in dimethyl sulfoxide (DMSO) and then diluting it in normal saline to make 4 mg/mL/kg BW in 1.6% DMSO/saline, and 10 mg/kg BW of ovalbumin was prepared for the positive control group in the same vehicle. The challenge was carried out by intravenously injecting the prepared solution with 1% Evans blue 24 h after immunization and the size of blue spots inside the skin was measured 30 min after the challenge. The formation of blue spots with a diameter larger than 5 mm was considered a positive cutaneous anaphylactic response.

### Statistical analysis

All data presented here are expressed as mean ± standard deviation. An SPSS software (Version 25, IBM) was employed for statistical analysis; Fisher’s exact test was used for *in vitro* chromosome aberration test, Mann–Whitney *U* test, and Kruskal–Wallis test followed by *post hoc* Tukey’s HSD multiple comparison test for *in vivo* micronucleus test. For all other studies, one-way ANOVA followed by *post hoc* Dunnett’s *t*-test was applied. A *p*-value less than 0.05 was considered statistically significant.

## Results

### Characterization of lac dye – Absorbance characteristics and quantification of laccaic acids

**FIGURE 1:**
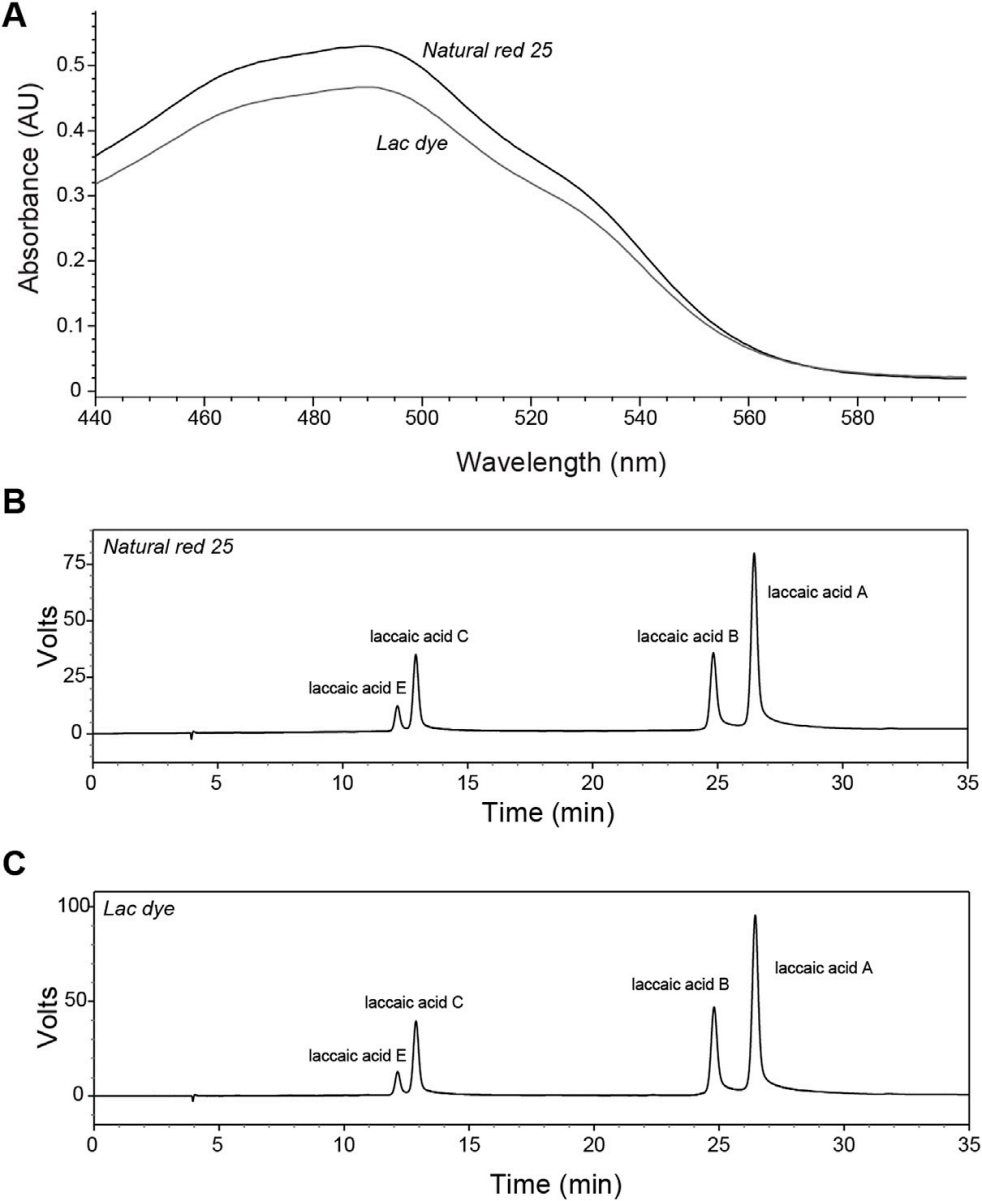
Characterization of lac dye by HPLC analysis. The test substance lac dye was analyzed using HPLC along with the reference compound natural red 25. **(A)** The absorbance patterns of the reference compound and lac dye were observed to be comparable with lower values detected for lac dye in the range of wavelength shorter than 560 nm. When laccaic acid isomers were compared, natural red 25 **(B)** and lac dye **(C)** showed high similarity in containing laccaic acid A as the most abundant isomer without laccaic acid D except for the difference in the ratio of isomer B and C in the test substance.

Lac dye is known to contain laccaic acid isomers, namely, laccaic acid A, B, C, D, and E, and, therefore, we carried out HPLC analysis on our test article to identify and quantify each laccaic acid isomer. The color value E (10%, 1 cm, 490 nm) was assessed to be 1512.19 for the reference compound NR25 and 1334.17 for LD. When the absorbance spectrum of LD was compared to that of NR25, both showed a closely similar pattern with its peak absorbance recorded at 490 nm: the absorbance of NR25 and LD at 490 nm were 0.530 and 0.467, respectively (Figure 1A). HPLC analysis found that NR25 and LD contained all laccaic acid isomers except for laccaic acid D with a slight difference in their proportion (Figures 1B,C); the ratios of laccaic acid isomers A, B, C, and E were 54.47%, 19.95%, 19.16%, and 6.42% (100% in total) for NR25 and 54.74%, 23.04%, 16.65%, and 5.56% (99.99%) for LD, respectively, indicating that unlike the almost identical ratios of isomer B and C in NR25, LD contained about 6.4% more isomer B than C. These results demonstrate that LD is highly pure and has the similar composition of laccaic acid isomers to the reference compound and the test substances used in a previous study (Hirata et al., 2001).

### Single-dose oral toxicity study

**FIGURE 2:**
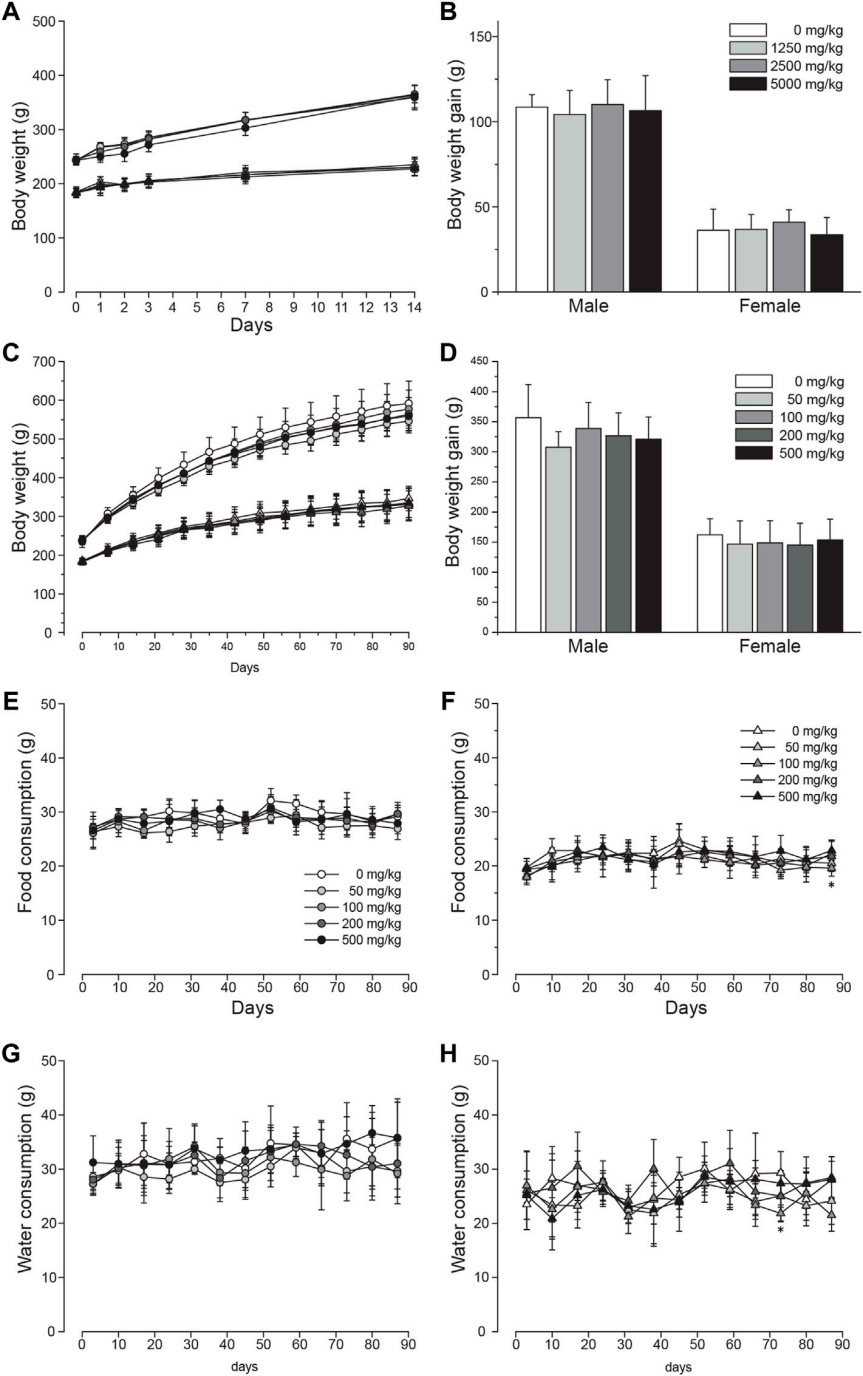
Unchanged body weight and food and water intake of SD rats orally administered with lac dye. Single oral administration of lac dye (LD) did not cause a recognizable change in **(A)** body weight and **(B)** body weight gain during the period of 14 days post administration. The animals orally administered LD for 90 days did not show differences in **(C)** body weight change and **(D)** body weight gain irrespective of treated doses. Circles are for male and triangles for female groups in **(A**,**C)** with symbol colors indicating the dose levels as described in **(B**,**D)**, respectively. The consumed amount of food (**E**; male and **F**; female) and water (**G**; male and **H**; female) was comparable among all of the groups during the 90-day test period. See the legends in **(E**,**F)** for the symbols used in **(G**,**H)**, respectively. *p < 0.05 by one-way ANOVA, followed by *post hoc* Dunnett’s *t*-test.

The systemic toxicity of LD was initially tested in a single-dose oral toxicity study. For the whole period of 14 days after single administration of either 0, 1250, 2500, or 5000 mg/kg BW, no animals were found with mortality or abnormal clinical signs. Body weight change was also comparable throughout the test (Figure 2A). Similarly, the body weight gain calculated after the study was found to be comparable among all groups (Figure 2B). At the terminal necropsy, no noticeable lesions related to the test substance were observed (data now shown). Based on these findings in the single dose toxicity study, the oral LD_50_ was determined to be >5000 mg/kg BW for both male and female SD rats.

### Repeated-dose oral toxicity study

We carried out a 28-day dose range finding study (0, 250, 500, 1000, 2000, and 5000 mg/kg BW) to discover appropriate dose levels for a 90-day repeated-dose toxicity study and selected 500 mg/kg BW as the suitable highest dose (data not shown). For the 90-day study, animals were orally administered either 50, 100, 200, or 500 mg/kg BW of LD, and the vehicle control groups had 10 ml/kg of distilled water. None of the treated animals were found dead during the study period. In male groups, salivation was observed in 3.9% ± 6.5 and 32.9% ± 8.7 of the animals in 200 and 500 mg/kg groups, respectively. The symptom started immediately after administration and usually continued for about 30 min. Also, hair loss was observed in one animal from week 11 in the male 100 and 500 mg/kg groups and in the female 100 mg/kg group. Low acidity of the prepared LD for administration was considered responsible for the dose-dependent occurrence of salivation (pH of 50 mg/ml; 2.56) and the hair loss was sporadic. There was no difference in the body weight change measured during the study period (Figure 2C) and body weight gain at the end of the study (Figure 2D), and generally consumption of food (Figure 2E for male groups and Figure 2F for female groups) and water (Figure 2G for male groups and Figure 2H for female groups) was not affected by the administration of the test substance with two exceptions that were considered incidental: the amount of food consumed in week 13 was significantly altered in female 100 mg/kg and water intake in week 11 in female 50, 100, and 200 mg/kg groups. Urinalysis (Supplementary Table S1) and ophthalmological examination (data not shown) performed on five animals per group in the last week did not identify any abnormality related to the test substance.

Hematological analysis performed at necropsy showed that all parameters examined were comparable across all groups regardless of the treated doses and substance (Table 1), while the levels of albumin in serum biochemistry were significantly elevated in the male 200 mg/kg group (Table 2). This change was considered incidental due to the lack of remarkable changes in other treated groups. Besides this, all other parameters in serum biochemical analysis remained unchanged in the treatment groups compared to the vehicle control group.

**TABLE 1:** Hematological parameters of SD rats orally treated with lac dye for 90 days.

	Dose of lac dye (mg/kg)				
	0	50	100	200	500
Male (*n* = 10/group)					
WBC (10^3^/mm^3^)	10.27 ± 1.85	10.54 ± 2.29	9.84 ± 2.66	8.69 ± 1.37	9.09 ± 1.71
RBC (10^6^/mm^3^)	8.36 ± 0.42	8.46 ± 0.23	8.29 ± 0.41	8.44 ± 0.42	8.17 ± 0.40
HGB (g/dl)	14.5 ± 0.4	14.8 ± 0.6	14.5 ± 0.8	14.7 ± 0.5	14.3 ± 0.5
HCT (%)	43.8 ± 1.4	44.3 ± 1.3	43.4 ± 2.1	43.7 ± 1.4	42.6 ± 1.2
PLT (10^3^/mm^3^)	1034.6 ± 122.5	1008.2 ± 95.3	962.9 ± 131.3	1009.2 ± 140.6	1044.9 ± 85.5
MCV (fl)	52.5 ± 1.6	52.4 ± 1.2	52.4 ± 1.6	51.9 ± 2.3	52.3 ± 1.7
MCH (pg)	17.4 ± 0.6	17.5 ± 0.5	17.5 ± 0.7	17.5 ± 0.9	17.5 ± 0.6
MCHC (g/dl)	33.2 ± 0.4	33.4 ± 0.5	33.5 ± 0.5	33.7 ± 0.7	33.4 ± 0.5
Neutrophils (%)	18.7 ± 11.6	15.5 ± 4.5	14.8 ± 2.7	17.4 ± 6.1	21.0 ± 5.2
Eosinophils (%)	0.9 ± 0.3	1.1 ± 0.5	1.1 ± 0.3	1.1 ± 0.4	1 ± 0.3
Basophils (%)	0.3 ± 0.1	0.2 ± 0.1	0.2 ± 0.1	0.2 ± 0.0	0.2 ± 0.1
Lymphocytes (%)	76.2 ± 11.9	80.1 ± 4.7	80.6 ± 3.0	77.9 ± 6.6	74.8 ± 5.2
Monocytes (%)	2.8 ± 0.8	2.2 ± 0.7	2.4 ± 0.9	2.6 ± 0.9	2.2 ± 0.7
Reticulocytes (%)	2.8 ± 1.0	2.6 ± 0.5	2.8 ± 0.5	2.7 ± 0.4	2.5 ± 0.4
PT (sec)	10.0 ± 0.9	10.7 ± 0.7	10.5 ± 0.8	10.1 ± 1	10.3 ± 0.7
aPTT (sec)	19.2 ± 2.0	19.1 ± 1.5	19.4 ± 1.6	18.4 ± 1.6	17.8 ± 1.9
					
Female (*n* = 10/group)					
WBC (10^3^/mm^3^)	8.38 ± 1.69	7.12 ± 1.56	7.14 ± 1.73	7.29 ± 1.43	6.46 ± 2.86
RBC (10^6^/mm^3^)	7.69 ± 0.32	7.6 ± 0.39	7.58 ± 0.28	7.52 ± 0.24	7.46 ± 0.33
HGB (g/dl)	14.2 ± 0.7	14.0 ± 0.5	14.3 ± 0.6	14.0 ± 0.3	14.0 ± 0.5
HCT (%)	41.7 ± 1.7	41.0 ± 1.5	41.6 ± 2.0	40.7 ± 1.1	40.5 ± 1.5
PLT (10^3^/mm^3^)	988.0 ± 84.3	979.4 ± 92.8	932.1 ± 101.8	957.8 ± 105.4	953.8 ± 116.8
MCV (fl)	54.2 ± 2.2	53.9 ± 1.8	54.9 ± 2.2	54.1 ± 1.8	54.4 ± 1.2
MCH (pg)	18.4 ± 0.8	18.4 ± 0.7	18.9 ± 0.6	18.6 ± 0.7	18.7 ± 0.5
MCHC (g/dl)	33.9 ± 0.6	34.2 ± 0.7	34.4 ± 0.7	34.3 ± 0.4	34.5 ± 0.4
Neutrophils (%)	11.2 ± 5.2	12.9 ± 3.7	10.7 ± 1.9	8.2 ± 1.9	13.5 ± 5.3
Eosinophils (%)	1.4 ± 0.6	1.2 ± 0.4	1.5 ± 0.4	1.3 ± 0.7	1.3 ± 0.5
Basophils (%)	0.2 ± 0.1	0.2 ± 0.0	0.2 ± 0.1	0.2 ± 0.1	0.2 ± 0.0
Lymphocytes (%)	83.5 ± 5.9	81.9 ± 4.4	84.0 ± 2.0	86.8 ± 2.8	81.2 ± 5.9
Monocytes (%)	2.6 ± 1.2	2.9 ± 1.1	2.5 ± 0.7	2.5 ± 0.9	2.9 ± 0.9
Reticulocytes (%)	2.2 ± 0.3	2.5 ± 0.5	2.6 ± 0.5	2.6 ± 0.6	2.4 ± 0.4
PT (sec)	8.6 ± 0.5	8.7 ± 0.4	8.5 ± 0.5	8.6 ± 0.3	8.6 ± 0.4
aPTT (sec)	19.4 ± 2.2	18.2 ± 2.3	18.5 ± 1.7	18.3 ± 1.6	19.3 ± 1.7

WBC, white blood cells; RBC, red blood cells; Hb, hemoglobin; HCT, hematocrit; PLT, platelet; MCV, mean corpuscular volume; MCH, mean corpuscular hemoglobin; MCHC, mean corpuscular hemoglobin concentration; PT, partial thromboplastin time; aPTT, activated partial thromboplastin time; BUN, blood urea nitrogen; TC, total cholesterol; TP, total protein; TB, total bilirubin; ALP, alkaline phosphatase; AST, aspartate aminotransferase (AST); ALT, alanine aminotransferase; GGT, γ-glutamyl transferase; and TG, triglycerides. *p < 0.05 by one-way ANOVA, followed by *post hoc* Dunnett’s *t*-test.

**TABLE 2:** Serum biochemical parameters of SD rats orally treated with lac dye for 90 days.

	Dose of lac dye (mg/kg)			
	0	50	100	200	500
Male (*n* = 10/group)					
BUN (mg/dl)	14.7 ± 2.0	14.4 ± 1.6	15.6 ± 3.8	13.9 ± 1.7	13.7 ± 2.4
TC (mg/dl)	84.0 ± 24.7	70.0 ± 15.1	74.8 ± 18.2	77.5 ± 16.7	82.3 ± 17.8
HDLC (mg/dl)	24.0 ± 4.5	22.3 ± 2.7	22.8 ± 3.7	23.5 ± 3.9	25.1 ± 4.1
LDLC (mg/dl)	9.0 ± 4.6	7.3 ± 1.5	7.1 ± 2.6	7.5 ± 1.8	8.1 ± 2.6
TP (g/dl)	5.6 ± 0.3	5.7 ± 0.3	5.6 ± 0.3	5.8 ± 0.2	5.7 ± 0.3
Albumin (g/dl)	2.2 ± 0.1	2.3 ± 0.1	2.2 ± 0.1	2.4 ± 0.1*	2.3 ± 0.1
TB (mg/dl)	0.1 ± 0.0	0.1 ± 0.0	0.1 ± 0.0	0.1 ± 0.0	0.1 ± 0.0
ALP (IU/L)	284.0 ± 51.0	330.6 ± 88.1	342.7 ± 145.6	369.9 ± 130.3	335.1 ± 107.0
AST (IU/L)	133.6 ± 69.7	115.9 ± 25.1	99.0 ± 17.9	105.8 ± 37.7	122.8 ± 47.9
ALT (IU/L)	50.5 ± 43.5	40.5 ± 6.5	36.5 ± 10.3	42.7 ± 19.4	46.3 ± 25.5
GGT (IU/L)	0.0 ± 0.0	0.0 ± 0.0	0.2 ± 0.4	0.0 ± 0.0	0.1 ± 0.3
Creatinine (mg/dl)	0.33 ± 0.09	0.33 ± 0.06	0.34 ± 0.04	0.30 ± 0.04	0.35 ± 0.04
TG (mg/dl)	82.1 ± 41.7	44.1 ± 13.1	62.0 ± 26.7	72.2 ± 32.3	52.4 ± 46.1
Glucose (mg/L)	151.0 ± 16.2	139.0 ± 15.4	150.3 ± 21.5	142.4 ± 17.0	144.9 ± 18.5
A/G	0.66 ± 0.05	0.68 ± 0.04	0.67 ± 0.05	0.70 ± 0.07	0.67 ± 0.05
Potassium (mEq/L)	4.6 ± 0.3	4.6 ± 0.3	4.6 ± 0.2	4.5 ± 0.2	4.4 ± 0.3
Chlorine (mEq/L)	103.2 ± 1.4	103.8 ± 0.9	103.6 ± 0.7	103.9 ± 0.7	104.6 ± 1.9
Sodium (mEq/L)	144.3 ± 0.7	144.5 ± 1.4	144.6 ± 0.8	145.0 ± 1.1	145.1 ± 1.2
Calcium (mg/dl)	9.6 ± 0.4	9.7 ± 0.3	9.7 ± 0.2	9.8 ± 0.3	9.6 ± 0.2
Phosphorous (mg/dl)	6.9 ± 0.5	7.0 ± 0.6	6.9 ± 0.5	6.8 ± 0.3	6.5 ± 0.6
					
Female (*n* = 10/group)					
BUN (mg/dl)	17.6 ± 3.1	17 ± 3.2	16.5 ± 3.5	16.5 ± 2.6	17.1 ± 2.2
TC (mg/dl)	102.6 ± 20.5	100.2 ± 30.4	105.7 ± 22.4	94.7 ± 14.5	104.5 ± 23.5
HDLC (mg/dl)	33.7 ± 5.8	33.2 ± 7.7	33.4 ± 3.7	32.1 ± 3.5	34.5 ± 6.3
LDLC (mg/dl)	6.1 ± 1.6	6.4 ± 2.0	8 ± 2.3	5.6 ± 0.8	6.7 ± 1.6
TP (g/dl)	6.5 ± 0.5	6.4 ± 0.4	6.5 ± 0.5	6.4 ± 0.3	6.6 ± 0.5
Albumin (g/dl)	2.9 ± 0.3	2.9 ± 0.2	3 ± 0.3	3 ± 0.3	3 ± 0.3
TB (mg/dl)	0.1 ± 0.0	0.1 ± 0.0	0.1 ± 0.0	0.1 ± 0.0	0.1 ± 0.0
ALP (IU/L)	246.5 ± 73.1	215.3 ± 42.7	276.3 ± 123.7	263.4 ± 101.4	215.2 ± 41.1
AST (IU/L)	123.1 ± 81.2	102.4 ± 17.1	103.2 ± 25.7	117.9 ± 57	108.5 ± 43.7
ALT (IU/L)	37.7 ± 11.4	38.5 ± 7.6	33.4 ± 3.3	43.7 ± 17.2	38.3 ± 14.6
GGT (IU/L)	0 ± 0.0	0.1 ± 0.3	0.1 ± 0.3	0 ± 0.0	0.1 ± 0.3
Creatinine (mg/dl)	0.37 ± 0.06	0.36 ± 0.07	0.33 ± 0.06	0.35 ± 0.07	0.35 ± 0.07
TG (mg/dl)	49.6 ± 23.4	42 ± 38.8	52.7 ± 18.1	44.4 ± 20.6	48.8 ± 47.4
Glucose (mg/L)	139.6 ± 15.0	147.1 ± 17.4	148.7 ± 19.2	149.4 ± 19.4	156 ± 15.9
A/G	0.83 ± 0.07	0.82 ± 0.06	0.85 ± 0.05	0.85 ± 0.05	0.83 ± 0.08
Potassium (mEq/L)	4.1 ± 0.3	4.2 ± 0.2	4.2 ± 0.3	4.2 ± 0.3	4.1 ± 0.4
Chlorine (mEq/L)	101.3 ± 1.8	101.8 ± 1.6	102.6 ± 1.0	102 ± 1.5	101.8 ± 2.4
Sodium (mEq/L)	143.3 ± 0.9	143.1 ± 0.9	143.4 ± 0.7	142.8 ± 1.5	142.8 ± 1.1
Calcium (mg/dl)	9.5 ± 0.4	9.5 ± 0.3	9.7 ± 0.2	9.5 ± 0.4	9.5 ± 0.4
Phosphorous (mg/dl)	5.8 ± 0.8	6.1 ± 0.5	6.3 ± 0.9	6.2 ± 0.9	5.7 ± 1.0

BUN, blood urea nitrogen; TC, total cholesterol; TP, total protein; TB, total bilirubin; ALP, alkaline phosphatase; AST, aspartate aminotransferase; ALT, alanine aminotransferase; GGT, γ-glutamyl transferase; and TG, triglycerides. *p < 0.05 by one-way ANOVA, followed by *post hoc* Dunnett’s *t*-test.

Measurement of organ weight (Table 3) showed that the weight of the liver was decreased in the LD-treated male groups with a significant reduction in the absolute weight detected in the 50 and 500 mg/kg groups and the relative weight in the male 50 and 100 mg/kg groups. Nevertheless, these changes were observed within the range of the institutional historic control data. In addition, the absolute weight of the left epididymis was markedly decreased in the 500 mg/kg group, while the relative weight of the right testis was increased in the 200 mg/kg group. Further analysis on sperm count, motility, and morphology did not detect abnormalities in the high-dose group compared to the vehicle control group (Supplementary Table S2). In female groups, the absolute weight of the brain and the relative weight of the pituitary gland were to a small degree but remarkably increased. Although statistically significant, lack of dose dependency and other dose groups with similar changes showed their incidental nature, negating an association with LD administration. The weight of parotid and submaxillary glands was comparable across all groups.

**TABLE 3:** Absolute and relative weight of major organs from SD rats orally treated with lac dye for 90 days.

		Dose of lac dye (mg/kg)										
			0		50			100		200		500	
Male (*n* = 10/group)													
Liver	(g)		16.52 ± 2.34		13.79 ± 0.77*			14.72 ± 1.88		15.02 ± 2.21		14.33 ± 1.42*	
	(g%)		2.89 ± 0.23		2.62 ± 0.15*			2.64 ± 0.19*		2.76 ± 0.26		2.67 ± 0.16	
Spleen	(g)		0.99 ± 0.22		0.88 ± 0.16			0.94 ± 0.14		0.90 ± 0.14		0.83 ± 0.06	
	(g%)		0.17 ± 0.03		0.17 ± 0.03			0.17 ± 0.02		0.17 ± 0.02		0.16 ± 0.01	
Kidney (R)	(g)		1.71 ± 0.17		1.62 ± 0.16			1.68 ± 0.21		1.67 ± 0.16		1.62 ± 0.17	
	(g%)		0.30 ± 0.03		0.31 ± 0.03			0.30 ± 0.03		0.31 ± 0.02		0.30 ± 0.04	
Kidney (L)	(g)		1.72 ± 0.21		1.61 ± 0.17			1.68 ± 0.21		1.66 ± 0.18		1.59 ± 0.17	
	(g%)		0.30 ± 0.03		0.31 ± 0.03			0.30 ± 0.03		0.31 ± 0.02		0.30 ± 0.03	
Adrenal gl. (R)	(g)		0.030 ± 0.005		0.029 ± 0.004			0.031 ± 0.006		0.027 ± 0.005		0.029 ± 0.003	
	(g%)		0.005 ± 0.001		0.006 ± 0.001			0.006 ± 0.001		0.005 ± 0.001		0.005 ± 0.001	
Adrenal gl. (L)	(g)		0.033 ± 0.005		0.031 ± 0.005			0.032 ± 0.005		0.030 ± 0.004		0.030 ± 0.004	
	(g%)		0.006 ± 0.001		0.006 ± 0.001			0.006 ± 0.001		0.006 ± 0.001		0.006 ± 0.001	
Testis (R)	(g)		1.75 ± 0.12		1.81 ± 0.21			1.77 ± 0.13		1.76 ± 0.07		1.85 ± 0.08	
	(g%)		0.31 ± 0.03		0.34 ± 0.04			0.32 ± 0.03		0.33 ± 0.03		0.35 ± 0.03*	
Testis (L)	(g)		1.81 ± 0.14		1.79 ± 0.20			1.80 ± 0.13		1.75 ± 0.08		1.90 ± 0.10	
	(g%)		0.32 ± 0.03		0.34 ± 0.04			0.33 ± 0.03		0.32 ± 0.03		0.36 ± 0.03	
Epididymis(R)	(g)		0.75 ± 0.05		0.75 ± 0.07			0.73 ± 0.08		0.73 ± 0.04		0.77 ± 0.09	
	(g%)		0.13 ± 0.01		0.14 ± 0.01			0.13 ± 0.02		0.14 ± 0.01		0.14 ± 0.02	
Epididymis(L)	(g)		0.78 ± 0.04		0.74 ± 0.08			0.72 ± 0.06		0.70 ± 0.03*		0.81 ± 0.07	
	(g%)		0.14 ± 0.01		0.14 ± 0.01			0.13 ± 0.01		0.13 ± 0.01		0.15 ± 0.02	
Prostate and Coagulating gl	(g)		3.52 ± 0.30		3.56 ± 0.33			3.46 ± 0.46		3.52 ± 0.41		3.60 ± 0.62	
	(g%)		0.62 ± 0.10		0.68 ± 0.07			0.62 ± 0.10		0.65 ± 0.09		0.67 ± 0.11	
Heart	(g)		1.67 ± 0.13		1.53 ± 0.09			1.65 ± 0.18		1.59 ± 0.17		1.59 ± 0.09	
	(g%)		0.29 ± 0.01		0.29 ± 0.02			0.30 ± 0.03		0.29 ± 0.02		0.30 ± 0.02	
Lung	(g)		1.68 ± 0.14		1.56 ± 0.10			1.70 ± 0.19		1.61 ± 0.10		1.61 ± 0.09	
	(g%)		0.30 ± 0.01		0.30 ± 0.02			0.31 ± 0.02		0.30 ± 0.02		0.30 ± 0.02	
Thymus	(g)		0.30 ± 0.05		0.24 ± 0.03			0.28 ± 0.07		0.26 ± 0.08		0.26 ± 0.05	
	(g%)		0.05 ± 0.01		0.05 ± 0.01			0.05 ± 0.01		0.05 ± 0.01		0.05 ± 0.01	
Thyroid (R)	(g)		0.015 ± 0.003		0.015 ± 0.003			0.016 ± 0.002		0.015 ± 0.003		0.015 ± 0.002	
	(g%)		0.003 ± 0.000		0.003 ± 0.000			0.003 ± 0.000		0.003 ± 0.001		0.003 ± 0.000	
Thyroid (L)	(g)		0.016 ± 0.003		0.015 ± 0.003			0.016 ± 0.003		0.015 ± 0.002		0.014 ± 0.002	
	(g%)		0.003 ± 0.000		0.003 ± 0.001			0.003 ± 0.001		0.003 ± 0.000		0.003 ± 0.000	
Brain	(g)		2.31 ± 0.13		2.21 ± 0.08			2.26 ± 0.12		2.22 ± 0.08		2.28 ± 0.09	
	(g%)		0.41 ± 0.03		0.42 ± 0.03			0.41 ± 0.03		0.41 ± 0.02		0.43 ± 0.03	
Pituitary gl	(g)		0.0143 ± 0.0016		0.0131 ± 0.0022			0.0136 ± 0.0018		0.0136 ± 0.0017		0.0133 ± 0.0011	
	(g%)		0.0025 ± 0.0004		0.0025 ± 0.0004			0.0025 ± 0.0004		0.0025 ± 0.0003		0.0025 ± 0.0002	
Parotid gl. (R)	(g)		0.16 ± 0.03		0.14 ± 0.02			0.15 ± 0.02		0.14 ± 0.02		0.14 ± 0.02	
	(g%)		0.03 ± 0.00		0.03 ± 0.00			0.03 ± 0.00		0.03 ± 0.00		0.03 ± 0.00	
Parotid gl. (L)	(g)		0.15 ± 0.03		0.14 ± 0.02			0.14 ± 0.02		0.14 ± 0.01		0.14 ± 0.01	
	(g%)		0.03 ± 0.01		0.03 ± 0.00			0.03 ± 0.00		0.03 ± 0.00		0.03 ± 0.00	
Submaxillary gl. (R)	(g)		0.41 ± 0.04		0.39 ± 0.03			0.40 ± 0.04		0.42 ± 0.04		0.41 ± 0.04	
	(g%)		0.07 ± 0.00		0.07 ± 0.00			0.07 ± 0.01		0.08 ± 0.01		0.08 ± 0.01	
Submaxillary gl. (L)	(g)		0.42 ± 0.03		0.39 ± 0.04			0.4 ± 0.04		0.41 ± 0.04		0.41 ± 0.03	
	(g%)		0.07 ± 0.00		0.07 ± 0.01			0.07 ± 0.01		0.08 ± 0.01		0.08 ± 0.01	
				0		50	100		200		500	
Female (*n* = 10/group)												
Liver		(g)		9.46 ± 1.14		8.87 ± 1.61	9.30 ± 0.86		9.23 ± 1.29		9.46 ± 1.12	
		(g%)		2.83 ± 0.35		2.72 ± 0.28	2.87 ± 0.27		2.90 ± 0.31		2.89 ± 0.20	
Spleen		(g)		0.57 ± 0.06		0.57 ± 0.08	0.58 ± 0.07		0.61 ± 0.10		0.56 ± 0.09	
		(g%)		0.17 ± 0.02		0.18 ± 0.03	0.18 ± 0.02		0.19 ± 0.03		0.17 ± 0.02	
Kidney (R)		(g)		1.02 ± 0.06		0.98 ± 0.11	0.98 ± 0.11		1.04 ± 0.09		1.03 ± 0.08	
		(g%)		0.31 ± 0.03		0.30 ± 0.02	0.30 ± 0.03		0.33 ± 0.04		0.31 ± 0.02	
Kidney (L)		(g)		0.98 ± 0.07		0.96 ± 0.11	0.94 ± 0.08		1.00 ± 0.08		1.01 ± 0.11	
		(g%)		0.29 ± 0.03		0.30 ± 0.02	0.29 ± 0.03		0.32 ± 0.03		0.31 ± 0.02	
Adrenal gl. (R)		(g)		0.034 ± 0.005		0.033 ± 0.004	0.033 ± 0.003		0.034 ± 0.004		0.035 ± 0.005	
		(g%)		0.010 ± 0.002		0.010 ± 0.002	0.010 ± 0.001		0.011 ± 0.002		0.011 ± 0.002	
Adrenal gl. (L)		(g)		0.037 ± 0.005		0.034 ± 0.004	0.034 ± 0.004		0.037 ± 0.004		0.037 ± 0.005	
		(g%)		0.011 ± 0.002		0.011 ± 0.002	0.011 ± 0.002		0.012 ± 0.002		0.011 ± 0.002	
Ovary (R)		(g)		0.040 ± 0.005		0.043 ± 0.010	0.039 ± 0.007		0.043 ± 0.008		0.034 ± 0.009	
		(g%)		0.012 ± 0.001		0.013 ± 0.003	0.012 ± 0.003		0.013 ± 0.002		0.010 ± 0.003	
Ovary (L)		(g)		0.041 ± 0.009		0.040 ± 0.011	0.042 ± 0.006		0.041 ± 0.011		0.036 ± 0.009	
		(g%)		0.012 ± 0.002		0.012 ± 0.003	0.013 ± 0.002		0.013 ± 0.003		0.011 ± 0.003	
Uterus		(g)		0.77 ± 0.23		0.74 ± 0.33	0.97 ± 0.66		0.75 ± 0.16		0.87 ± 0.36	
		(g%)		0.23 ± 0.06		0.24 ± 0.12	0.30 ± 0.22		0.24 ± 0.07		0.27 ± 0.12	
Heart		(g)		1.06 ± 0.11		0.99 ± 0.09	1.03 ± 0.10		1.04 ± 0.08		1.00 ± 0.08	
		(g%)		0.32 ± 0.02		0.31 ± 0.03	0.32 ± 0.03		0.33 ± 0.03		0.31 ± 0.03	
Lung		(g)		1.28 ± 0.09		1.28 ± 0.09	1.30 ± 0.10		1.32 ± 0.14		1.29 ± 0.16	
		(g%)		0.38 ± 0.04		0.40 ± 0.05	0.40 ± 0.04		0.42 ± 0.08		0.40 ± 0.06	
Thymus		(g)		0.27 ± 0.09		0.23 ± 0.07	0.27 ± 0.09		0.27 ± 0.08		0.24 ± 0.06	
		(g%)		0.08 ± 0.02		0.07 ± 0.02	0.08 ± 0.03		0.09 ± 0.02		0.07 ± 0.02	
Thyroid (R)		(g)		0.012 ± 0.002		0.013 ± 0.004	0.012 ± 0.003		0.013 ± 0.003		0.013 ± 0.002	
		(g%)		0.004 ± 0.001		0.004 ± 0.001	0.004 ± 0.001		0.004 ± 0.001		0.004 ± 0.001	
Thyroid (L)		(g)		0.011 ± 0.002		0.011 ± 0.002	0.013 ± 0.002		0.012 ± 0.003		0.011 ± 0.001	
		(g%)		0.003 ± 0.001		0.003 ± 0.001	0.004 ± 0.001		0.004 ± 0.001		0.003 ± 0.001	
Brain		(g)		1.95 ± 0.10		2.05 ± 0.09	2.03 ± 0.07		2.07 ± 0.09*		1.99 ± 0.10	
		(g%)		0.59 ± 0.06		0.64 ± 0.07	0.63 ± 0.06		0.65 ± 0.08		0.61 ± 0.06	
Pituitary gl		(g)		0.0181 ± 0.0024		0.0201 ± 0.0044	0.0174 ± 0.0038		0.0204 ± 0.0024		0.0220 ± 0.0050	
		(g%)		0.0054 ± 0.0008		0.0062 ± 0.0010	0.0054 ± 0.0012		0.0065 ± 0.0011		0.0068 ± 0.0017*	
Parotid gl. (R)		(g)		0.12 ± 0.02		0.12 ± 0.02	0.12 ± 0.02		0.12 ± 0.03		0.12 ± 0.02	
		(g%)		0.04 ± 0.01		0.04 ± 0.01	0.04 ± 0.01		0.04 ± 0.01		0.04 ± 0.01	
Parotid gl. (L)		(g)		0.12 ± 0.02		0.11 ± 0.02	0.12 ± 0.02		0.13 ± 0.03		0.12 ± 0.02	
		(g%)		0.04 ± 0.00		0.04 ± 0.01	0.04 ± 0.01		0.04 ± 0.01		0.04 ± 0.01	
Submaxillary gl. (R)		(g)		0.25 ± 0.03		0.23 ± 0.02	0.25 ± 0.03		0.25 ± 0.02		0.25 ± 0.03	
		(g%)		0.07 ± 0.01		0.07 ± 0.01	0.08 ± 0.01		0.08 ± 0.01		0.08 ± 0.01	
Submaxillary gl. (L)		(g)		0.25 ± 0.02		0.24 ± 0.03	0.25 ± 0.03		0.25 ± 0.02		0.25 ± 0.03	
		(g%)		0.07 ± 0.01		0.07 ± 0.01	0.08 ± 0.01		0.08 ± 0.01		0.08 ± 0.01	

*p < 0.05 by one-way ANOVA, followed by *post hoc* Dunnett’s *t*-test.

Gross observation of organs during necropsy identified regional color changes including discoloration and red and brown spots in the liver, lung, thymus, cervical lymph node, and ovaries at similar frequencies in the vehicle and LD-treated groups (Supplementary Table S3). It is to be noted that a red–purple pigmentation was observed on the surface of the skull and femur in 50% male and 30% female animals from the 500 mg/kg group, but not in any other LD-treated or vehicle control groups. The pigmentation was considered transient in nature as it was washed off upon submerging into the fixative and also undetected in the recovery group (data not shown). Histopathological analysis of the major organs for microscopic lesions revealed a minimal to moderate degree of inflammation in several organs including salivary glands, liver, pancreas, thymus, spleen, and cervical lymph node, but there was no recognizable difference in its frequency and extent between the vehicle and LD-treated groups (Supplementary Table S4), suggesting that such lesions were not related to administration of the test substance. It is noteworthy that no lesions were found in association with the pigmentation observed in gross observation. Taken together, these findings demonstrated that oral administration of LD did not cause adverse effects in the tested species, establishing that NOAEL in SD rats is 500 mg/kg BW.

### Mutagenicity of lac dye

**FIGURE 3:**
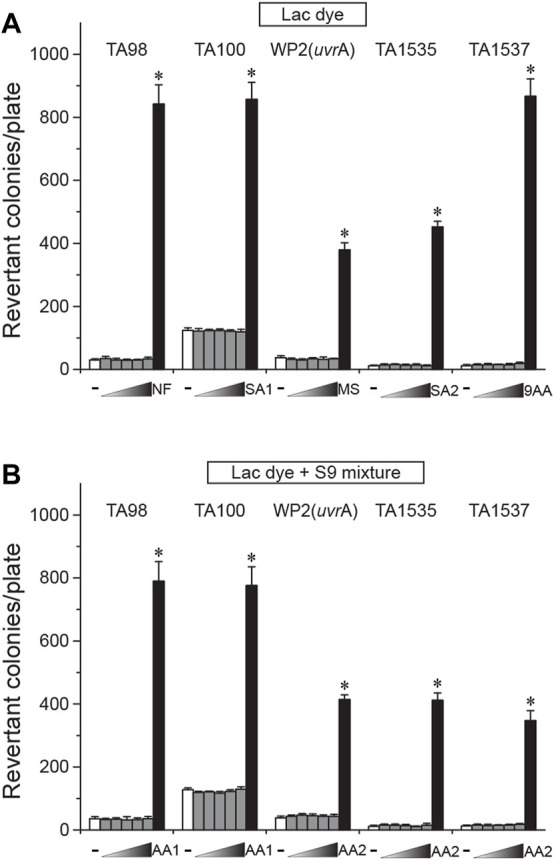
Lack of mutagenicity in lac dye shown by the bacterial reverse mutation test. Lac dye (LD) was treated on four *S. typhimurium* and one *E. coli* tester strains for assessment of mutagenicity. Up to 5000 μg/plate of LD did not increase the number of revertant colonies regardless of metabolic activation by the S-9 mixture, while the respective positive controls resulted in a significant increase. White bars, vehicle control; gray bars, lac dye (312.5, 625, 1250, 2500, and 5000 μg/plate from the left); and black bars, positive controls (NF, 10.0 μg/plate 2-nitrofluorene; SA1, 5.0 μg/plate sodium azide; MS, 2.5 μg/plate methylmethane sulfonate; SA2, 0.5 μg/plate sodium azide; 9AA, 80.0 μg/plate 9-aminoacridine; AA1, 2.0 μg/plate 2-aminoanthracene; and AA2, 5.0 μg/plate 2-aminoanthracene). Negative signs indicate the vehicle control, and right-angled triangles with gradient shade, increasing doses of LD. *p < 0.05 by one-way ANOVA, followed by *post hoc* Dunnett’s *t*-test.

Next, we assessed the genotoxicity of LD using the reverse bacterial mutation test. Before the main test, we confirmed that LD did not elicit cytotoxicity up to 5000 µg/plate using the two *S. typhimurium* tester strains TA100 and TA1537 (data not shown). When treated on four *S. typhimurium* (TA98, TA100, TA1535, and TA1537) and one *E. coli* (WP2*uvrA*) tester strains, LD did not increase the number of revertant colonies in the tester strains at any doses compared to the vehicle control regardless of S-9 mixture co-treatment, while the respective positive controls resulted in a marked increase (Figure 3). These results indicated that LD does not have mutagenic potential in the bacterial tester strains.

### *In vitro* clastogenicity of lac dye

**FIGURE 4:**
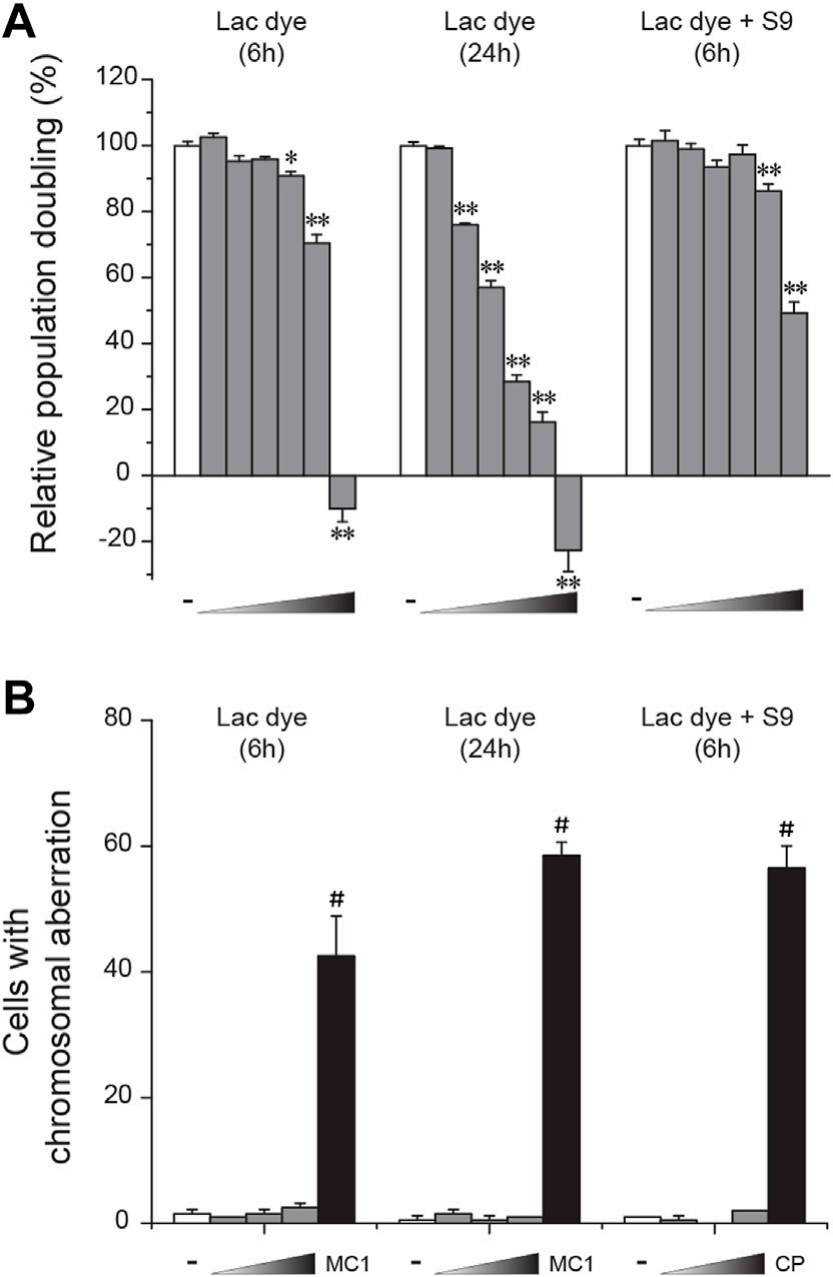
Lack of *in vitro* clastogenicity in lac dye. Lac dye (LD) was tested for clastogenicity using Chinese hamster lung (CHL) cells. **(A)** When tested for cytotoxicity using relative population doubling, LD showed higher cytotoxicity by increasing incubation time while metabolic activation resulted in a marked reduction. **(B)** The selected sublethal doses for each condition (375, 750, and 1500 μg/ml for 6-h treatment; 125, 250, and 500 μg/ml for 24-h; and 625, 1250, and 2500 μg/ml for 6-h with S-9 mixture) did not increase the number of cells containing aberrant chromosomes, while the positive control group showed a significant increase. White bars, vehicle control; gray bars, lac dye; and black bars, positive controls (MC1, 0.1 μg/ml mitomycin C and CP, 5.0 μg/ml cyclophosphamide). Negative signs indicate the vehicle control, and right-angled triangles with gradient shade, increasing doses of LD. *p < 0.05 when compared to the control by Fisher’s exact.

CHL cells were used to test *in vitro* clastogenicity of LD. When cytotoxicity was examined, LD caused a significant reduction of relative population doubling from 625 μg/ml in the 6-h treatment group, and from as low as 156.3 μg/ml in the 24-h treatment group (Figure 4A), indicating a dose- and time-dependent increase of cytotoxicity. Interestingly, metabolic activation with co-treatment of the S-9 mixture was found to increase the degree of LD-induced cytotoxicity. When the impact of LD on chromosomes was assessed by treating CHL cells with a sublethal and two lower doses selected from the cytotoxicity test, the numbers of cells containing aberrant chromosomes in the LD-treated groups were similar to those in the respective vehicle groups (Figure 4B), demonstrating the absence of *in vitro* clastogenicity in LD. On the contrary, the positive control group significantly increased the number, ensuring the validity of the test. It is to be noted that the frequency of structural abnormality in the chromosomes also remained unchanged in the LD-treated groups (data not shown).

### *In vivo* clastogenicity of lac dye

**FIGURE 5:**
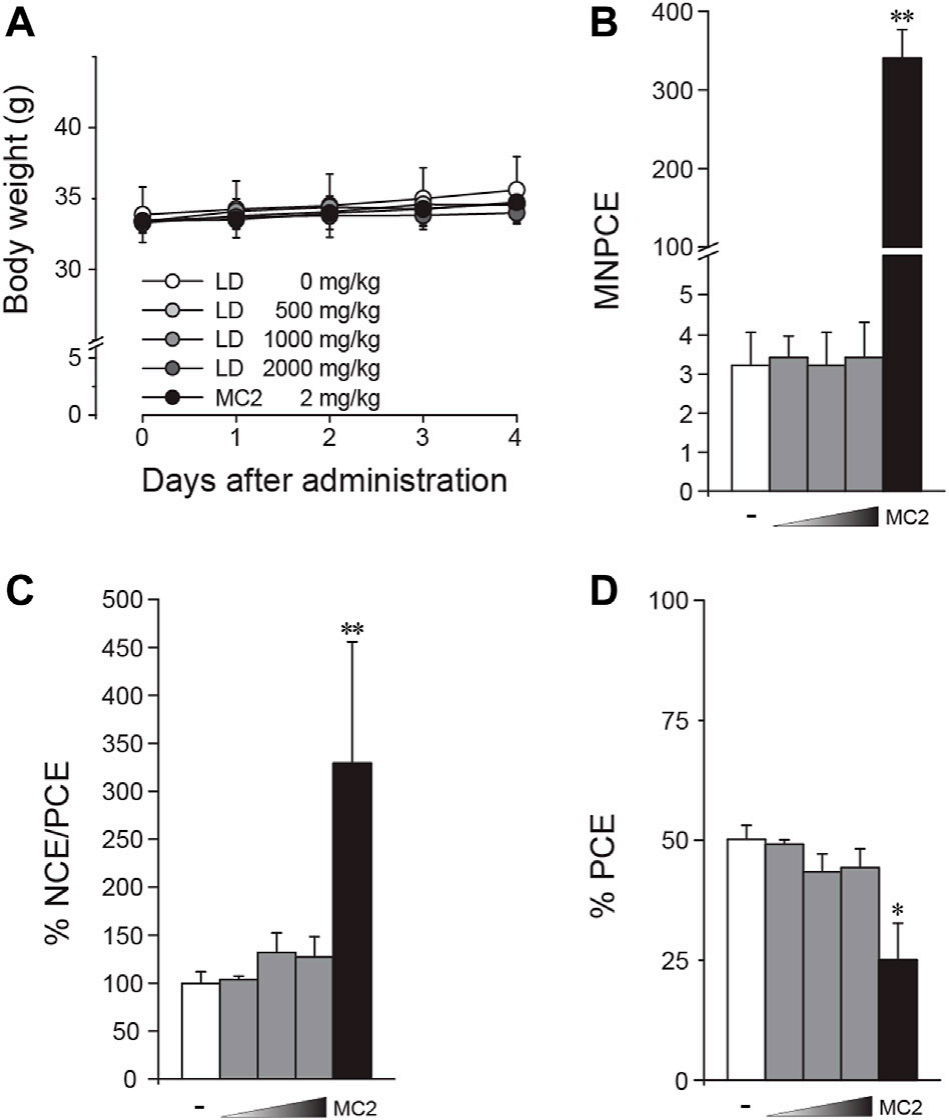
Absence of *in vivo* clastogenicity in lac dye. Eight-week-old male mice were treated with lac dye (LD) for 3 days, and the number of micronucleus-containing polychromatic erythrocytes (MNPCEs) from bone marrow cells isolated from the femora was counted for assessment of clastogenicity. **(A)** Mice treated with LD did not show differences in body weight change compared to the vehicle control group. LD did not cause an increase in **(B)** MNPCE, **(C)** % NCE/PCE, and **(D)** % PCE compared to the vehicle control group, while the positive control induced a significant change. White bars, vehicle control; gray bars, lac dye (500, 1000, and 2000 mg/kg from the left); and black bars, positive controls (MC2, 2 mg/kg mitomycin) **(C)**. Negative signs indicate the vehicle control, and right-angled triangles with gradient shade, increasing doses of LD. *p < 0.05 by the Kruskal–Wallis test, followed by *post hoc* Tukey’s HSD multiple comparison test.

Following the *in vitro* tests, an *in vivo* genotoxicity of LD was tested through formation of micronuclei in SD rat bone marrow cells. After confirming that the high dose of 2000 mg/kg BW was well tolerated, the mice were orally administered up to 2000 mg/kg BW once for 3 days, and bone marrow cells were prepared from the femora for microscopic observation. During the test period, there was no change in body weight observed (Figure 5A). Compared to results from the vehicle groups, LD treatment did not induce significant deviation in the number of MNPCEs (Figure 5B), the ratio of NCE/PCE (Figure 5C) and the ratio of PCE (Figure 5D), while the positive control mitomycin C induced significant changes in all of the parameters. Together with the *in vitro* results, these results suggested the lack of *in vivo* clastogenic potentials in LD.

### *In vivo* antigenicity of lac dye

Despite its insect origin and potential action as an allergen, the impact of lac dye on the immune system remained unexplored. Here, we performed a passive cutaneous anaphylaxis test to assess the antigenicity of LD. For challenge *via* intravenous injection, LD, unlike normal saline used for oral administration, was completely dissolved in 1.6% DMSO. During the period of sensitization, the average body weights of all groups were similar regardless of treatment (data not shown). When challenged after dermal injection of serially diluted immunization serum into SD rats, the positive group showed an Evan blue positive spot in the subcutaneous area of injection between 2^6^- and 2^1^-fold dilution, indicating successful induction of cutaneous anaphylactic responses using ovalbumin (Table 4). On the contrary, all LD-treated groups including LD conjugated with FCA did not result in the appearance of the Evan blue spots, suggesting that LD lacks antigenicity.

**TABLE 4:** Frequency of positive response in the passive cutaneous anaphylaxis test on lac dye.

Anti-serum dilution factor	Dose of lac dye (mg/kg)							Ovalbumin (mg/kg)
	0	0.5	1	2	4	4 + FCA		5 + FCA
2^10^	0/10	0/10	0/10	0/10	0/10	0/10		0/10
2^9^	0/10	0/10	0/10	0/10	0/10	0/10		0/10
2^8^	0/10	0/10	0/10	0/10	0/10	0/10		0/10
2^7^	0/10	0/10	0/10	0/10	0/10	0/10		0/10
2^6^	0/10	0/10	0/10	0/10	0/10	0/10		2/10
2^5^	0/10	0/10	0/10	0/10	0/10	0/10		6/10
2^4^	0/10	0/10	0/10	0/10	0/10	0/10		10/10
2^3^	0/10	0/10	0/10	0/10	0/10	0/10		10/10
2^2^	0/10	0/10	0/10	0/10	0/10	0/10		10/10
2^1^	0/10	0/10	0/10	0/10	0/10	0/10		10/10

FCA, Freund’s complete adjuvant.

## Discussion

Lac dye has been used as an orange–red food colorant in diverse kinds of food products, and its use in the food industry has been growing due to consumers’ preference of natural color ingredients over synthetic counterparts. Previously, the safety of lac dye was assessed in a diet-mixed formulation, allowing its toxic effects to be investigated when used in solid food. In the present study, we formulated LD into aqueous suspension and systematically evaluated its general toxicity with a concurrent assessment of genotoxic potential as a food additive to beverage products. In addition, we attempted to assess the antigenicity of LD using an *in vivo* anaphylaxis model.

The test substance used in the study was analyzed to determine the proportion of each laccaic acid, and HPLC results showed that chemical characteristics of LD, albeit minor differences, had a high level of similarity to the reference compound NR25; the dominant isomer was laccaic acid A accounting for more than 50%, and D isomer was not detected in either compound. On the contrary, LD contained more isomer B than C, while the reference compound contained similar levels. These results are closely in line with the previous study, which through the analysis of six lac dye products found a subtle difference in the ratios of isomers B and C with a consistent absence of isomer D (Hirata et al., 2001) and suggested a difference in the origin of raw material and extraction methods as potential causes of the discrepancy observed in composition between lac dye products.

Single-dose oral administration of the chemically defined test substance showed that the highest dose of LD did not cause mortality or toxicity-related changes, establishing that oral LD_50_ in rats is >5000 mg/kg BW. Consistently, it was previously shown that 200 mg/kg BW of lac dye did not cause acute toxicity when fed in a diet (Thombare et al., 2017). Although the formulation of lac dye was unclear, no toxicity-related signs were reported in the albino rats fed with 2000 mg/kg BW during the 14-day observation period (Srivastava et al., 2013). Another group also reported a lack of hepatotoxicity and nephrotoxicity in mice fed with 1400 mg/kg BW of olive oil-dissolved lac dye (Kawazoe et al., 2000). In line with these, our findings also showed no acute toxicity at doses up to 5000 mg/kg BW suspended in water, expanding the safe range of lac dye.

A 90-day repeated-dose toxicity study was performed based on the findings from the acute study and the separate dose-range finding study. During the study period, transient salivation was observed in a dose-dependent manner at the high range of LD doses, but there were no toxicity-related pathological lesions observed in the salivary glands of these animals. Unlike our findings, Sakamoto et al. reported, without clinical signs for excessive salivation, enlargement and diffuse hypertrophy of acinar cells in the parotid gland accompanying ultrastructural changes in the animals fed with a diet containing 5% lac dye from a 13-week study (Sakamoto et al., 1998; Fukumori et al., 2000) with additional involvement of submaxillary gland in the female group in the chronic study (Sakamoto et al., 2000). These changes were considered as an adaptive nonadverse effect caused by unknown stimuli from the diet containing lac dye as also demonstrated in a rodent oral study on a grape skin extract-containing diet based on the reversal of parotid gland hypertrophy after cessation of administration (Inoue et al., 2013; Inoue et al., 2014). Acidic solution is known to induce enlargement of salivary glands with salivation (Cox et al., 1957) and the solution containing lac dye had low pH; pH 3.82 for 0.343 mg/ml (Kongkachuichay et al., 2002) and pH 2.56 for 50 mg/ml in this study. Therefore, acidic lac dye seemed to function as a sialotrophic stimulus to induce excessive salivation in our study as well as the histological changes observed in the previous study. Currently, the cause of the differences between the studies by Sakamoto et al. and ours is unknown, but the difference in the formulation of the test substance and resulting extent of exposure in the oral cavity may have influenced as a contributing factor. Based on these findings, excessive salivation observed in the LD-treated animals is likely an adaptive response caused by incidental exposure to the acidic LD in the oral cavity during gavage.

The formulation method may have differentially affected *in vivo* behavior of lac dye. The previous studies reported that animals fed with high doses of lac dye displayed dark purple pigmentation on the root of tongue without accompanying histological changes (Sakamoto et al., 1998; Sakamoto et al., 2000) and increased mineralization in kidneys in some animals. Interestingly, we also found red–purple pigmentation in a portion of animals from the high-dose-treated groups but in different parts of the body, including the surface of the skull and femur, and there was no evidence for mineralization in the kidney. Although considered unrelated to adverse effects based on its transient nature and lack of supportive evidence in histopathology, a further study investigating the effect of formulation of lac dye on its toxicokinetics is warranted to elucidate the underlying mechanism of the observed differences.

The results of the *in vivo* passive cutaneous anaphylaxis test indicated a lack of antigenicity under the conditions of the current study. Although the validity of the test was confirmed, limitations associated with the use of DMSO should be noted for the application and interpretation of these results. In the study, 1.6% DMSO was used to completely dissolve LD for intravenous administration at the challenge stage. DMSO has been shown to function as an immunosuppressant by reducing autoimmune antibody titers in several model systems (Pestronk and Drachman, 1980; Weetman et al., 1982) and has also recently demonstrated to dose-dependently alleviate the severity of anaphylactic shock and extend the time to mortality in horse serum-sensitized guinea pigs (Semyonov et al., 1995). Furthermore, the observation that the positive control ovalbumin dissolved in the same vehicle elicited a detectable response at 2^6^-fold dilution, which is 4–8-fold lower than those at which the positive responses appeared in other studies that we performed, corroborated that DMSO possibly weakened, if any, the immune response caused by LD. Normal appearance of immune-related organs in the LD-treated animals in the present study and a previous report on the suppressing effect of micromolar lac dye (DMSO-dissolved and treated at 0.1%) on the secretion of IgE from rat spleen lymphocytes (Kuramoto et al., 1996) support our findings on the lack of antigenicity, but further investigation using different model systems may be required to verify these results.

Finally, we showed that LD did not have mutagenicity nor clastogenicity through the results of genotoxicity tests and no cancerous legion was observed in the repeated-dose toxicity study. These results were highly consistent with the previous findings from the genotoxicity and carcinogenicity studies (Banerjee et al., 1984; Dube et al., 1984), providing another line of evidence to support its lack of genotoxicity.

In this study, we systematically evaluated the oral safety of lac dye by carrying out studies for general toxicity, genotoxicity, and antigenicity. Our results demonstrated that LD did not cause recognizable toxicity in rats in the acute and subacute studies nor genotoxicity in the battery of *in vitro* and *in vivo* tests. In addition, we reported for the first time direct evidence for lack of antigenicity by assessing its potential to trigger anaphylactic responses *in vivo*. Collectively, these findings extended our knowledge on the safety of lac dye as a food colorant, providing a safety guideline for human use, especially as an additive to beverage products.

## Conclusion

Safety assessment of lac dye by the single-dose and 90-day repeated-dose oral toxicity studies showed that LD did not cause test substance-associated toxicity in SD rats, establishing its LD_50_ and NOAEL to be >5000 mg/kg BW and 500 mg/kg BW, respectively. A combination of *in vitro* and *in vivo* genotoxicity tests revealed that LD was not mutagenic nor clastogenic. In addition, LD was not causally linked to antigenicity in an *in vivo* model system. These findings broadened the safety profile of lac dye as a coloring additive to food and beverages, facilitating its safe use for human consumption. Nonetheless, caution should be taken for interpretation and application of the results presented in this study due to the limitation associated with the tests and potential toxicity caused by unknown interactions of lac dye with other food ingredients.

## Data Availability

The raw data supporting the conclusions of this article will be made available by the authors, without undue reservation.
