# Supplementary material for: Nonclinical safety evaluation of food colorant lac dye via systematic toxicity profiling with assessment of in vivo antigenic potential
Source: Front Pharmacol. 2022 Oct 31;13:1020379. doi: 10.3389/fphar.2022.1020379 (PMC9662299; doi:10.3389/fphar.2022.1020379)
Supplement: Supplementary file 1 [file Table1.pdf]

Supplementary Table 1. Urinalysis of SD rats orally treated with Lac dye for 90 days

Parameter	Value	Dose of Lac dye (mg/kg)									
		Male (n=5/group)					Female (n=5/group)				
		0	50	100	200	500	0	50	100	200	500
Specific gravity		1.044 ±0.006	1.037 ±0.009	1.038 ±0.013	1.036 ±0.008	1.027 ±0.013	1.034 ±0.015	1.026 ±0.022	1.031 ±0.009	1.028 ±0.014	1.033 ±0.013
Glucose (mg/dl)	negative	5/5	5/5	5/5	5/5	5/5	5/5	5/5	5/5	5/5	5/5
Bilirubin	negative	4/5	5/5	5/5	5/5	5/5	5/5	4/5	5/5	5/5	5/5
	small	1/5	0/5	0/5	0/5	0/5	0/5	1/5	0/5	0/5	0/5
Ketone	negative	1/5	0/5	0/5	0/5	1/5	3/5	3/5	4/5	4/5	2/5
(mg/dl)	trace	0/5	1/5	5/5	1/5	3/5	1/5	2/5	1/5	1/5	3/5
	15	3/5	4/5	0/5	4/5	1/5	1/5	0/5	0/5	0/5	0/5
	40	1/5	0/5	0/5	0/5	0/5	0/5	0/5	0/5	0/5	0/5
Blood	negative	1/5	5/5	3/5	2/5	2/5	5/5	5/5	5/5	4/5	5/5
	tract-intact	3/5	0/5	2/5	2/5	3/5	0/5	0/5	0/5	1/5	0/5
	trace-lysed	1/5	0/5	0/5	1/5	0/5	0/5	0/5	0/5	0/5	0/5
pH	6.5	0/5	0/5	0/5	0/5	0/5	1/5	1/5	1/5	0/5	0/5
	7.0	1/5	0/5	3/5	1/5	1/5	1/5	0/5	1/5	0/5	0/5
	7.5	2/5	2/5	0/5	1/5	2/5	1/5	0/5	1/5	1/5	2/5
	8.0	2/5	1/5	1/5	2/5	2/5	2/5	2/5	0/5	0/5	0/5
	8.5	0/5	2/5	1/5	1/5	0/5	0/5	2/5	2/5	4/5	3/5
Protein	negative	0/5	0/5	0/5	0/5	1/5	1/5	3/5	1/5	3/5	2/5
(mg/dl)	trace	0/5	2/5	1/5	0/5	2/5	2/5	0/5	3/5	1/5	1/5
	30	3/5	3/5	3/5	5/5	2/5	2/5	1/5	1/5	1/5	2/5
	100	2/5	0/5	1/5	0/5	0/5	0/5	0/5	0/5	0/5	0/5
	300	0/5	0/5	0/5	0/5	0/5	0/5	1/5	0/5	0/5	0/5
Urobilinogen	0.2	3/5	4/5	4/5	5/5	4/5	4/5	3/5	4/5	4/5	3/5
(E.U./dl)	1	2/5	1/5	1/5	0/5	1/5	1/5	2/5	1/5	1/5	2/5
Nitrite	negative	5/5	5/5	5/5	5/5	5/5	5/5	4/5	5/5	5/5	5/5
	positive	0/5	0/5	0/5	0/5	0/5	0/5	1/5	0/5	0/5	0/5
Leukocytes	negative	2/5	2/5	2/5	2/5	3/5	3/5	4/5	4/5	5/5	3/5
(cells/µL)	trace	3/5	3/5	3/5	3/5	2/5	2/5	1/5	1/5	0/5	2/5

Supplementary table 2. Sperm measures of SD rats orally treated with Lac dye for 90 days

Parameter	Dose of Lac dye (mg/kg)	
	Male (n=10/group)	
	0	500
Organ weight		
Necropsy body weight (g)	570.4 ± 57.7	536.3 ± 35.3
Testis (g)	1.81 ± 0.14	1.9 ± 0.1
Epididymis (g)	0.78 ± 0.04	0.81 ± 0.07
Cauda epididymis (g)	0.36 ± 0.02	0.36 ± 0.05
Sperm counts		
Sperm count (10^7/ g testis)	0.95 ± 0.17	0.89 ± 0.08
Sperm count (10^7/ testis)	1.71 ± 0.31	1.7 ± 0.19
Motile sperm (%)	94.2 ± 4.3	94.3 ± 3.7
Sperm morphology		
Abnormal sperms/200 sperms	7.1 ± 2.9	9.1 ± 3.7

Supplementary table 3. Gross findings in major organs from SD rats orally treated with Lac dye for 90 days

Organ	Findings	Dose of Lac dye (mg/kg)									
		Male (n=10/group)					Female (n=10/group)				
		0	50	100	200	500	0	50	100	200	500
Liver	Normal	9/10	10/10	10/10	10/10	9/10	9/10	10/10	10/10	10/10	9/10
	Discoloration	0/10	0/10	0/10	0/10	0/10	0/10	0/10	0/10	0/10	1/10
	Brown spot	1/10	0/10	0/10	0/10	1/10	1/10	0/10	0/10	0/10	0/10
Lung	Normal	10/10	10/10	10/10	9/10	9/10	9/10	9/10	10/10	9/10	10/10
	Redness	0/10	0/10	0/10	0/10	0/10	1/10	0/10	0/10	0/10	0/10
	Discoloration	0/10	0/10	0/10	0/10	0/10	0/10	0/10	0/10	1/10	0/10
	Red spot	0/10	0/10	0/10	1/10	0/10	0/10	1/10	0/10	0/10	0/10
	Brown spot	0/10	0/10	0/10	0/10	1/10	0/10	0/10	0/10	0/10	0/10
Thymus	Normal	8/10	10/10	8/10	10/10	10/10	9/10	8/10	6/10	10/10	8/10
	Redness	2/10	0/10	2/10	0/10	0/10	1/10	2/10	2/10	0/10	2/10
	Red spot	0/10	0/10	0/10	0/10	0/10	0/10	0/10	2/10	0/10	0/10
Cervical lymph node	Normal	4/10	7/10	7/10	6/10	7/10	9/10	3/10	6/10	5/10	5/10
	Redness	6/10	3/10	3/10	4/10	3/10	1/10	7/10	4/10	5/10	5/10
Skull	Normal	10/10	10/10	10/10	10/10	5/10	10/10	10/10	10/10	10/10	7/10
	Red-purple pigmentation	0/10	0/10	0/10	0/10	5/10	0/10	0/10	0/10	0/10	3/10
Femur	Normal	10/10	10/10	10/10	10/10	5/10	10/10	10/10	10/10	10/10	7/10
	Red-purple pigmentation	0/10	0/10	0/10	0/10	5/10	0/10	0/10	0/10	0/10	3/10
Ovary	Normal	-	-	-	-	-	9/10	10/10	9/10	9/10	9/10
	Redness	-	-	-	-	-	0/10	0/10	1/10	1/10	1/10
	Black spot	-	-	-	-	-	1/10	0/10	0/10	0/10	0/10
Clitoral glands	Normal	-	-	-	-	-	10/10	10/10	10/10	10/10	9/10
	Brown spot	-	-	-	-	-	0/10	0/10	0/10	0/10	1/10

Supplementary Table 4. Histopathological findings in major organs of SD rats orally treated with Lac dye for 90 days

Organ	Findings	Dose of Lac dye (mg/kg) Male (n=10/group) 0	Male 500	Female (n=10/group) 0	Female 500
Nervous system					
Brain	NAD	10/10	10/10	10/10	10/10
Spinal cord	NAD	10/10	10/10	10/10	10/10
Sciatic nerve	NAD	10/10	10/10	10/10	10/10
Ocular system					
Eyes	NAD	10/10	10/10	10/10	10/10
Harderian glands	NAD	8/10	5/10	7/10	9/10
	Focal minimal MCI, unilateral	0/10	5/10	3/10	1/10
	Focal minimal MCI, bilateral	1/10	0/10	0/10	0/10
	Diffuse mild MCI, bilateral	1/10	0/10	0/10	0/10
Digestive system					
Tongue/larynx	NAD	7/10	6/10	9/10	8/10
	Focal minimal MCI (tongue)	1/10	0/10	0/10	1/10
	Focal minimal MCI (larynx)	2/10	3/10	1/10	1/10
	Focal minimal MCI (tongue and larynx)	0/10	1/10	0/10	0/10
Salivary glands	NAD	2/10	3/10	9/10	8/10
	Focal minimal MCI, unilateral (sublingual)	1/10	2/10	1/10	1/10
	Focal minimal MCI, bilateral (sublingual)	3/10	1/10	0/10	0/10
	Diffuse minimal MCI, unilateral (sublingual)	0/10	1/10	0/10	0/10
	Diffuse minimal MCI, bilateral (sublingual)	3/10	2/10	0/10	1/10
	Diffuse mild MCI, bilateral (sublingual)	1/10	1/10	0/10	0/10
Esophagus	NAD	10/10	10/10	10/10	10/10
Stomach	NAD	10/10	10/10	10/10	10/10
Duodenum	NAD	10/10	10/10	10/10	10/10
Jejunum	NAD	10/10	10/10	10/10	10/10
Ileum	NAD	10/10	10/10	10/10	10/10
Cecum	NAD	10/10	10/10	10/10	10/10
Colon	NAD	10/10	10/10	10/10	10/10
Rectum	NAD	10/10	10/10	10/10	10/10
Liver	NAD	1/10	0/10	0/10	0/10
	Focal minimal MCI	0/10	0/10	0/10	2/10
	Diffuse minimal MCI	8/10	8/10	8/10	8/10
	Diffuse mild MCI	0/10	1/10	1/10	0/10
	Diffuse mild MHN; diffuse minimal MCI	1/10	0/10	1/10	0/10
	Diffuse mild MHN; diffuse minimal microvesicular fatty change	0/10	1/10	0/10	0/10
Pancreas	NAD	9/10	8/10	8/10	7/10
	Focal minimal MCI	1/10	2/10	0/10	3/10
	Focal mild MCI	0/10	0/10	1/10	0/10
	Diffuse minimal MCI	0/10	0/10	1/10	0/10
Immune system					
Thymus	NAD	3/10	9/10	7/10	4/10
	Ultimobranchial cyst	4/10	0/10	0/10	1/10
	Diffuse minimal hemorrhage	1/10	0/10	2/10	4/10
	Diffuse mild hemorrhage	2/10	1/10	1/10	1/10
Spleen	NAD	10/10	10/10	10/10	10/10
Cervical lymph node	NAD	5/10	5/10	9/10	8/10
	Diffuse minimal congestion	3/10	4/10	1/10	2/10
	Diffuse mild congestion	2/10	1/10	0/10	0/10
Mesenteric lymph node	NAD	10/10	10/10	10/10	0/10
Endocrine system					
Pituitary gland	NAD	10/10	10/10	10/10	10/10
Thyroid glands /	NAD	8/10	6/10	7/10	6/10
Parathyroid glands	Ultimobranchial cyst	1/10	4/10	3/10	4/10
	Focal minimal MCI	1/10	0/10	0/10	0/10
Adrenal glands	NAD	6/10	5/10	8/10	10/10
	Diffuse minimal cortical vacuolization	4/10	3/10	0/10	0/10
	Diffuse mild cortical vacuolization	0/10	2/10	0/10	0/10
	Minimal cystic degeneration	0/10	0/10	1/10	0/10
	Mild cystic degeneration	0/10	0/10	1/10	0/10

MCI, mononuclear cell infiltrate; MHN, midzonal hepatocellular necrosis; NAD, no abnormalities detected

Supplementary Table 4. Histopathological findings in major organs from SD rats orally treated with Lac dye for 90 days (continued)

Organ	Findings	Dose of Lac dye (mg/kg)			
		Male (n=10/group)		Female (n=10/group)	
		0	500	0	500
Respiratory system					
Nasal cavity	NAD	10/10	9/10	10/10	10/10
	Diffuse severe SI	0/10	1/10	0/10	0/10
Trachea	NAD	10/10	10/10	10/10	10/10
Lung	NAD	10/10	10/10	10/10	10/10
Cardiovascular system					
Heart	NAD	7/10	4/10	10/10	7/10
	Focal minimal MCI	1/10	5/10	0/10	3/10
	Diffuse mild MCI	1/10	0/10	0/10	0/10
	Mild cardiomyopathy with fibrosis; diffuse mild MCI	1/10	0/10	0/10	0/10
	Moderate cardiomyopathy with fibrosis; diffuse moderate MCI	0/10	1/10	0/10	0/10
Urinary system					
Kidneys	NAD	6/10	3/10	6/10	4/10
	Focal minimal MCI	0/10	1/10	0/10	0/10
	Focal minimal MCI, unilateral	2/10	5/10	4/10	5/10
	Focal minimal bilateral	2/10	1/10	0/10	0/10
	Focal minimal MCI, bilateral; minimal calcification, unilateral	0/10	0/10	0/10	1/10
Urinary bladder	NAD	10/10	10/10	9/9*	10/10
Reproductive system					
Preputial gland / Clitoral glands	NAD	7/10	10/10	10/10	9/10
	Focal minimal MCI, unilateral	1/10	0/10	0/10	1/10
	Focal minimal MCI, unilateral; focal minimal SI, unilateral	1/10	0/10	0/10	0/10
	Focal minimal MCI, unilateral; focal mild SI, unilateral	1/10	0/10	0/10	0/10
Testes	NAD	10/10	10/10	-	-
Epididymides	NAD	10/10	10/10	-	-
Prostate	NAD	2/10	1/10	-	-
	Focal minimal MCI (ventral lobe)	1/10	1/10	-	-
	Focal minimal SI (lateral lobe)	0/10	1/10	-	-
	Focal mild MCI (ventral lobe)	0/10	1/10	-	-
	Diffuse minimal MCI (ventral lobe)	3/10	2/10	-	-
	Diffuse mild MCI (ventral lobe)	2/10	2/10	-	-
	Diffuse mild MCI; focal minimal SI (lateral lobe)	0/10	1/10	-	-
	Diffuse mild MCI; focal minimal SI (ventral lobe)	1/10	0/10	-	-
	Diffuse moderate MCI (ventral lobe)	1/10	1/10	-	-
Seminal vesicle	NAD	10/10	10/10	-	-
Ovaries	NAD	-	-	10/10	10/10
Uterus	NAD	-	-	10/10	10/10
Vagina	NAD	-	-	10/10	10/10
Musculoskeletal and other systems					
Skin/mammary gland	NAD	10/10	10/10	10/10	10/10
Skeletal muscle	NAD	10/10	10/10	10/10	10/10
Sternum	NAD	10/10	10/10	10/10	10/10
Femur / bone marrow	NAD	10/10	10/10	10/10	10/10

**, Total number of organs prepared and examined is shown*

NAD, no abnormalities detected; MCI, mononuclear cell infiltrate; SI, suppurative inflammation
